# Efficiacy of Pharmacotherapy in Patients with Hypothimic Mental Disorders Suffered from Covid-19 Infection

**DOI:** 10.1192/j.eurpsy.2023.435

**Published:** 2023-07-19

**Authors:** D. Labunskiy, D. Kuzmin, D. Baranov, S. Kiryukhina, V. Pavelkina

**Affiliations:** ^1^Ogarev Mordovia State University, Saransk; ^2^Vidnoe District Hospital, Vidnoe, Russian Federation

## Abstract

**Introduction:**

In organic mental disorders in people who have undergone COVID-19, it has been established that the complex use of periciazine in combination with paroxetine, diazepam, 3-hydroxypyridine succinate and hyperbaric oxygenation is superior in effectiveness to traditional therapy with an antipsychotic drug, antidepressant and anxiolytic. The inclusion of 3-hydroxypyridine succinate, hyperbaric oxygenation in the complex therapy of this pathology corrects the concentrations of adrenaline, norepinephrine, dopamine, serotonin in the peripheral blood of patients, eliminates hormonal status disorders and humoral immune responses.

**Objectives:**

The aim of the work was to optimize approaches to the treatment of hypothymic disorders in organic mental illness, to substantiate the complex use of periciazine in combination with paroxetine, diazepam, 3-hydroxypyridine succinate and hyperbaric oxygenation in patients who underwent COVID-19.

**Methods:**

The object of the clinical study were patients with organic mental disorders who underwent COVID-19. To assess the condition, laboratory research methods were selected taking into account the etio- and pathogenesis of diseases: determining the level of catecholamines, some indicators of humoral immune responses, and the hormonal profile.

**Results:**

Table 2.3 - Nosological structure of patients included in the study

**Table:** 

Nosological form	Associated hypothymic disorder	Number of patients	Gender	Average age (years)	
			Males		Females
Organic mental disorder	Organic anxiety disorder F06.4	21	15	6	28,7±6,3
	Depressive Episode F33	22	12	9	
Organic mental disorder associated with COVID-19	Organic anxiety disorder F06.4	18	15	3	
	Depressive Episode F33	16	10	6	43,7±7,4

**Conclusions:**

In patients with organic mental disorders, occurring with hypothymic symptoms, compared with healthy donors, there is a complex of disorders in plasma concentrations of catecholamines. Traditional and, to a greater extent, combination therapy increase the levels of serotonin, dopamine, norepinephrine, both in the group of patients who did not have COVID-19, and in those who underwent a new coronovirus infection.

In patients with organic mental disorders, occurring with hypothymic symptoms, compared with healthy donors, there is a complex of disorders in plasma concentrations of catecholamines. Traditional and, to a greater extent, combination therapy increase the levels of serotonin, dopamine, norepinephrine, both in the group of patients who did not have COVID-19, and in those who underwent a new coronovirus infection.Complex therapy with periciazine, paroxetine, diazepam in combination with 3-hydroxypyridine succinate and HBO for organic mental disorders causes a more complete reduction of hypothymic disorders both in the group of patients who did not have COVID-19, and in those who underwent a new coronovirus infection.

**Disclosure of Interest:**

None Declared

